# Examining normative values using the Cambridge neuropsychological test automated battery and developmental traits of executive functions among elementary school-aged children in Japan

**DOI:** 10.3389/fpsyg.2023.1141628

**Published:** 2023-08-16

**Authors:** Sho Aoki, Fumiyo Nagatani, Kuriko Kagitani-Shimono, Yuko Ohno, Masako Taniike, Ikuko Mohri

**Affiliations:** ^1^Department of Child Development, United Graduate School of Child Development, Osaka University, Osaka, Japan; ^2^Molecular Research Center for Children's Mental Development, United Graduate School of Child Development, Osaka University, Osaka, Japan; ^3^Department of Pediatrics, Osaka University Graduate School of Medicine, Osaka, Japan; ^4^Department of Health Sciences, Osaka University Graduate School of Medicine, Osaka, Japan

**Keywords:** spatial working memory, planning, attentional set shifting and flexibility, inhibition, neuropsychological assessment

## Abstract

The Cambridge Neuropsychological Test Automated Battery (CANTAB) is a computerized and child-friendly neuropsychological assessment battery that includes subtests aimed at evaluating some aspects of executive functions. Using the CANTAB, this study aims to establish normative values based on the aspects of executive functions among school-aged children in Japan. The participants included 234 children (135 boys and 99 girls aged 6–12 years) enrolled in regular classes, without any clinical records of developmental disorders or educational support. The participants were grouped according to age (6–7, 8–9, and 10–12 years). Four CANTAB subtests, including spatial working memory (SWM) to assess spatial working memory, Stockings of Cambridge (SOC) to evaluate planning, intra/extradimensional set shift (IED) to evaluate attentional set shifting and flexibility, and stop signal task (SST) to evaluate inhibition, were administered to each participant. The results showed that performance in all the CANTAB subtests administered changed with age. Among the subtests, compared with performances in the SOC and IED, those in the SWM and SST improved earlier, thereby indicating that spatial working memory and inhibition develop earlier than planning as well as attentional set shifting and flexibility. Additionally, in the SST subtest, girls made fewer errors than boys did in the 6–7 years group. This study presents normative data of four CANTAB subtests according to age and sex among school-aged children in Japan. We expect that the findings will be used to develop effective tools for the early detection of and support for children with executive dysfunction.

## 1. Introduction

Executive functions (EFs) are broadly defined as cognitive processes that mediate goal-directed behavior, and they are organized based on neuronal activity, which is mainly mediated by the prefrontal cortex (PFC) (Best and Miller, 2010). In the related literature, EFs are postulated to incorporate the following components: inhibition that stops or overrides a mental process intentionally or unintentionally (MacLeod, 2007) and working memory that temporarily stores and manipulates the information necessary for complex cognitive tasks (Baddeley, 1992). Other components include cognitive flexibility to selectively switch between mental processes and further generate appropriate behavioral responses (Dajani and Uddin, 2015); and planning to design and evaluate a series of future actions (Nitschke et al., 2017). Although they show different developmental trajectories, these components are also considered inter-related and interdependent (Anderson, 2002).

The development of EFs is inferred to improve significantly during school age, and it continues until adolescence and early adulthood (Anderson, 2002; Romine and Reynolds, 2005). School age is the period during which the PFC, which is one of the cortical areas to develop last, matures (Fuster, 2002), and whereby experiences as well as individual differences during early childhood appear to have observable effects (Best et al., 2009). EFs are closely related to every aspect, such as scholarly success and behavioral problems, of the daily lives of school-aged children (Gathercole et al., 2004; Borella et al., 2010; Zorza et al., 2016; Bathelt et al., 2018). Therefore, it is crucial for people handling school-aged children to understand EFs within a developmental context.

The Cambridge Neuropsychological Test Automated Battery (CANTAB) is a computerized neuropsychological assessment battery that was originally developed for the assessment of cognitive functions among the elderly (Robbins et al., 1994, 1998). The CANTAB uses a touchscreen computer, and it includes tasks that assess some aspects of EFs. The CANTAB is considered easily applicable to children because the format is interesting and motivating, and all the task stimuli are nonverbal (Luciana, 2003). For example, this test has been used to evaluate impaired EFs among children with neurodevelopmental disorders, including autism spectrum disorders (Corbett et al., 2009; Yerys et al., 2009; Chen S. F. et al., 2016) and attention deficit hyperactivity disorder (Corbett et al., 2009; Nagatani et al., 2012; Coghill et al., 2014). Moreover, normative data for children have been collected in various countries, such as the USA (Luciana and Nelson, 2002), Australia (De Luca et al., 2003), Brazil (Roque et al., 2011), and Mexico (Green et al., 2019). A previous review has reported that East Asian and Caucasian children from North America and Europe have shown differences in the development of EFs and have been subject to different cultural and socialization influences (Cho et al., 2023). It has also been reported that cultural variation in the gray matter volume of the PFC is related to the dopamine D4 receptor gene (Yu et al., 2019). Thus, practically speaking, although it is certainly essential to obtain normative data from community samples of children of different ages and sexes, to evaluate the performance of various clinical groups and those who have maladjustments (Robbins et al., 1994; Luciana and Nelson, 2002), reference data for Japanese children are yet to been collected.

This study aims to establish normative values and consider the effect of age and sex on some aspects of EFs among school-aged children in Japan, as evaluated by the CANTAB. This study provides information regarding the different developmental trajectories of each component of EFs during school age among children in Japan, and it contributes to the early detection of and provision of support for children with executive dysfunction. Additionally, a preliminary investigation was conducted to examine the differences between the outcomes of the sample in this study and that of previous study (Luciana and Nelson, 2002), which might suggest referring to normative values suitable for children.

## 2. Methods

### 2.1. Participants

The participants were recruited through public newsletters distributed in Osaka Prefecture and from elementary schools in Osaka Prefecture. Children with a clinical history of neurological or neurodevelopmental disorders or those who had received special educational support were excluded from this study. The final sample used for the current analyses included 234 children aged 6–12 years, comprising 135 boys [mean ± standard deviations (SD); 8.74 ± 1.73 years] and 99 girls (8.58 ± 1.60 years). The sample size was determined based on previous studies (Luciana and Nelson, 2002; De Luca et al., 2003).

Written informed consent for participation, in accordance with the principles outlined in the Declaration of Helsinki, was obtained from all the participants and their guardians. This study was approved by the Institutional Review Board of Osaka University Hospital (#12168-9).

### 2.2. Tasks

**Figure 1:**
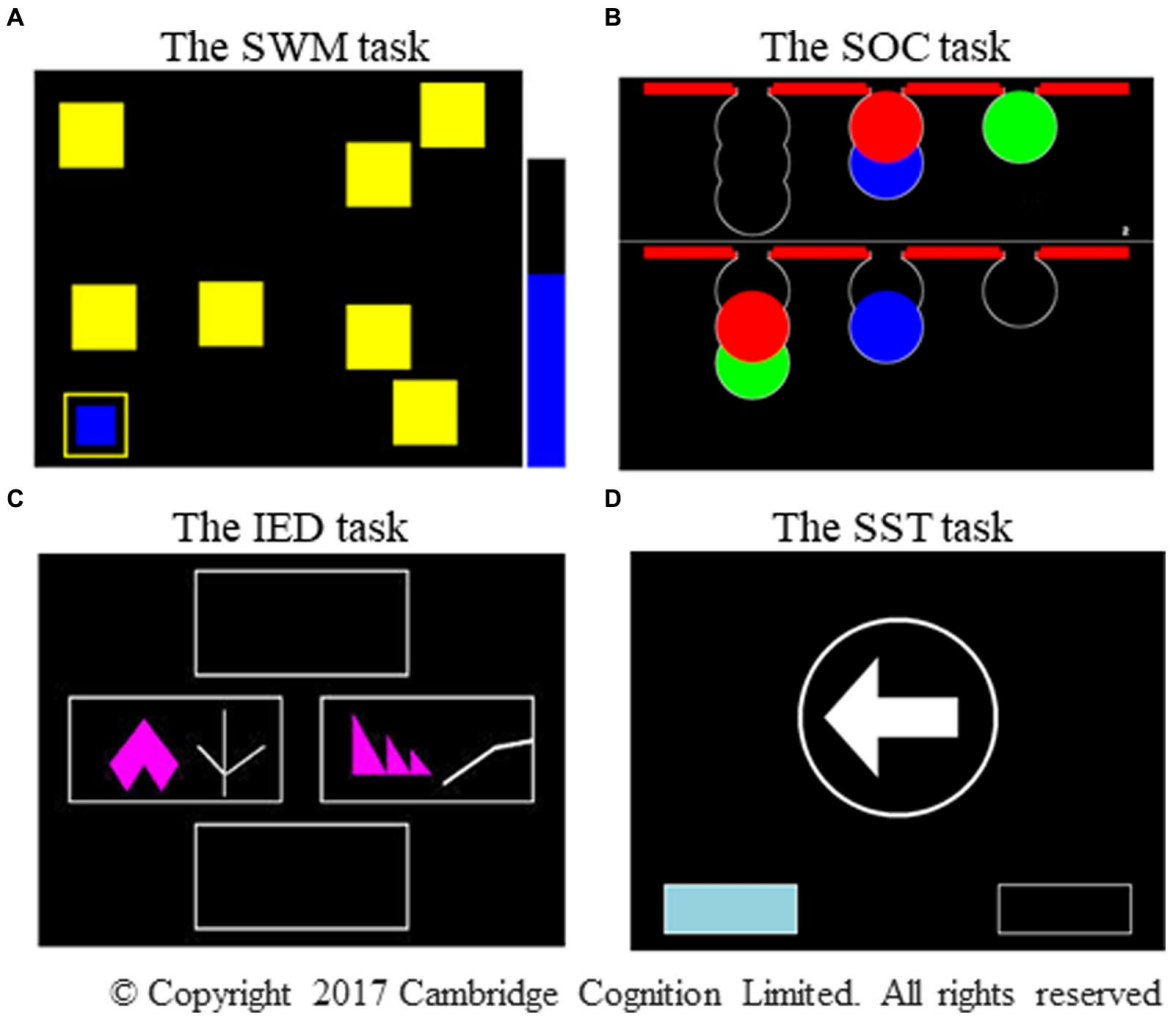
Photographs of the four CANTAB subtests. The photographs represent **(A)** the SWM task, **(B)** the SOC task, **(C)** the IED task, and **(D)** the SST subtest, respectively. SWM, spatial working memory; SOC, Stockings of Cambridge; IED, intra/extradimensional set shift; SST, stop signal task. Adapted with permission from Cambridge Cognition.

Four CANTAB^1^ subtests, including Spatial Working Memory (SWM), Stockings of Cambridge (SOC), Intra/ Extradimensional Set Shift (IED), Stop Signal Task (SST) were administered via a computer with a touch-enabled monitor (Figure 1). The administration of these four subtests lasted for approximately 50 min. Considering the motivation to perform, relatively easy tasks (SWM/SST) were followed by relatively difficult tasks (SOC/IED). In cases where it was difficult to complete all the four subtests depending on the age and stamina of the child, only two subtests (SWM and SST) were administered.

#### 2.2.1. Spatial working memory

This self-ordered task assesses spatial working memory and heuristic strategies (Owen et al., 1990). The procedure involves finding a blue token hidden in each of the boxes and placing it into an empty column on the right-hand side of the screen (Figure 1A). No blue token was found in any box where it had been previously located. This task became increasingly difficult as the number of boxes increased from three to eight. This study presents two key outcome measures: between errors in n boxes (the number of times a participant revisited a box where a blue token had already been found on the four-, six-, and eight-box problems) and strategy (the number of times a participant began a new search with a different box on the six- and eight-box problems). A high score on between errors reflected poor spatial working memory, and a high score on strategy reflected poor use of the strategy.

#### 2.2.2. Stockings of Cambridge

The SOC task, based on the traditional Tower of London, was used to evaluate planning (Owen et al., 1990). This task required moving the balls in the lower display for a predetermined number of times (two to five moves) to make it similar to the pattern shown in the upper display (Figure 1B). The variable of interest involved the number of problems solved in minimum moves (the number of times a participant perfectly completed a test problem using the minimum possible number of moves). A high score on the problems solved using the minimum moves possible reflected good planning ability.

#### 2.2.3. Intra/extradimensional set shift

The IED task is an adaptation of the Wisconsin card-sorting test, which was designed to assess attentional set shifting and flexibility (Owen et al., 1991). The participants were required to form a correct response using feedback provided automatically by the computer. The task, which comprises nine stages, began with simple discrimination between two color-filled shapes displayed on the screen, and it ended with the shift of attention to a novel exemplar of a previously irrelevant perceptual dimension (extradimensional shift: EDS, Figure 1C). The criterion of set formation proceeding to the next stage was when a participant satisfied six consecutive correct responses at each stage. The test was terminated in cases where a participant failed to meet this criterion after 50 trials at a particular stage. Two outcome measures were recorded: stages completed, which was the total number of stages a participant completed successfully, and EDS errors, which was the number of errors that occurred in the EDS stage. Low scores on the stages completed and high scores on EDS errors reflected poor attentional set shifting and flexibility.

#### 2.2.4. Stop signal task

The SST subtest is a classic signal response inhibition test (Curley et al., 2018). The participants pressed the relevant button on the press pad as quickly as possible, depending on the direction to which the arrow pointed on the screen (go trials, Figure 1D). However, if an auditory signal was present at a variable delay after the arrow was displayed, the participants withheld their responses and did not press the button (stop trials). This task comprised go trials (75%) and stop trials (25%) presented over five blocks of 64 trials each. The outcome measures were direction errors and proportion of successful stops. The former counted the number of times a participant pressed the wrong button for the direction of the arrow on the screen in the stop and go trials, and the latter referred to the number of times a participant stopped successfully, divided by the total number of stop signals using all the assessed trials. A high score on direction errors and a low proportion of successful stops reflected poor impulse control.

### 2.3. Data analysis

The participants were divided into three groups according to age, such as 6–7, 8–9, and 10–12 years. Because of larger inter-individual differences, especially among younger children, and the custom of dividing 6 years of elementary school into low, middle, and high grades from qualitative changes in the learning curriculum, which requires increased levels of abstract thinking, we divided our sample into three age groups on a two-year basis. In the SST subtest, the participants who showed noncompliance with the instructions or were characterized by more than two SD of the mean reaction time on the go trials when the correct button was pressed were excluded from this analysis.

We investigated the distributions of scores based on the visual inspection of histograms and the Shapiro–Wilk normality test. Considering the effect of age, we first examined the main effect of age. We further conducted *post-hoc* analyses to test the differences between each age group. To address the effect of sex, sex differences within each age group were also examined.

Two-way analysis of variance, with age group and sex as the between-subject factors, was used to compare the between errors in the eight-box problem in the SWM. The proportion of successful stops in the SST subtest was assessed using a two-way analysis of covariance, with age group and sex as the between-subject factors and the mean reaction time on the go trials of the SST subtest as a covariate. We performed the Kruskal-Wallis test for the between errors in the four- and six-box problems as well as strategy in the SWM, the problems solved using the minimum moves possible in the SOC, both outcome measures in the IED, and direction errors in the SST subtest because skewed distributions were observed.

The level of significance was set at *p* < 0.05. In the *post-hoc* analyses applying Bonferroni’s correction, the corrected *p*-value was calculated in the parametric test. However, because the uncorrected *p* value was provided in the non-parametric test, the *p* value was manually corrected and set at *p* < 0.017.

Spearman rank-correlation coefficients were calculated to examine interrelationships among variables of the tasks. The level of significance was set at *p* < 0.001.

Additionally, in the preliminary investigation examining the differences between the outcomes of this study’s sample and that of the previous study, we first adjusted our data to correspond to the age range shown in the previous study, such as six, seven, eight, 9–10, and 11–12 years (Luciana and Nelson, 2002). We used the summary independent-samples Welch’s *t*-test for the outcome measures common in both studies, which are as follows: between errors in the four-, six-, and eight-box problems and strategy scores in the SWM, the problems solved using the minimum possible moves in the SOC, and the stages completed in the IED. The level of significance was manually corrected and set at *p* < 0.01 to allow for the application of Bonferroni’s correction.

All the analyses were performed using SPSS version 22.0 (IBM, Tokyo, Japan).

## 3. Results

Table 1 presents the number of participants, mean score, and SD of each index in task performance within each sex-segregated age group. Table 1 also presents the median and range in the case of non-normal distribution (indicated by ^※^).

**Table 1:** Number, mean, and SD of each index in task performance.

(A) SWM						
Age		*N*	Between errors (4-box)	Between errors (6-box)	Between errors (8-box)	Strategy
6–7	(Boy)	42	2.45 ± 2.56	14.05 ± 7.90	32.33 ± 8.00	37.05 ± 3.32
			2.00 (0–10)^※^	15.50 (0–26)^※^		37.00 (29–42)^※^
	(Girl)	31	1.77 ± 2.62	15.32 ± 6.66	32.35 ± 8.89	37.29 ± 5.20
			0.00 (0–10)^※^	16.00 (2–26)^※^		38.00 (22–46)^※^
	Total	73	2.16 ± 2.59	14.59 ± 7.38	32.34 ± 8.33	37.15 ± 4.19
			1.00 (0–10)^※^	16.00 (0–26)^※^		37.00 (22–46)^※^
8–9	(Boy)	45	1.84 ± 1.92	12.58 ± 6.82	28.56 ± 11.48	35.73 ± 3.96
			2.00 (0–8)^※^	13.00 (1–26)^※^		36.00 (23–43)^※^
	(Girl)	40	1.73 ± 3.56	10.90 ± 8.57	26.13 ± 12.59	35.25 ± 4.41
			0.00 (0–17)^※^	8.00 (1–35)^※^		36.00 (25–43)^※^
	Total	85	1.79 ± 2.80	11.79 ± 7.69	27.41 ± 12.01	35.51 ± 4.16
			1.00 (0–17)^※^	11.00 (1–35)^※^		36.00 (23–43)^※^
10–12	(Boy)	48	0.58 ± 1.62	5.63 ± 5.25	16.92 ± 12.10	32.15 ± 4.91
			0.00 (0–8)^※^	4.00 (0–22)^※^		32.50 (19–40)^※^
	(Girl)	28	1.32 ± 2.54	7.61 ± 7.35	20.07 ± 10.24	33.79 ± 5.55
			0.00 (0–9)^※^	5.00 (0–26)^※^		35.00 (19–48)^※^
	Total	76	0.86 ± 2.03	6.36 ± 6.13	18.08 ± 11.48	32.75 ± 5.18
			0.00 (0–9)^※^	4.00 (0–26)^※^		34.50 (19–48)^※^

(B) SOC			
Age		*N*	Problems solved in minimum moves
6–7	(Boy)	32	6.16 ± 1.57
			6.00 (3–10)^※^
	(Girl)	17	6.47 ± 1.51
			7.00 (4–9)^※^
	Total	49	6.27 ± 1.54
			6.00 (3–10)^※^
8–9	(Boy)	34	6.35 ± 1.56
			6.00 (3–11)^※^
	(Girl)	28	6.96 ± 1.60
			6.50 (4–10)^※^
	Total	62	6.63 ± 1.59
			6.00 (3–11)^※^
10–12	(Boy)	41	7.66 ± 2.00
			7.00 (3–12)^※^
	(Girl)	25	7.60 ± 1.53
			8.00 (5–10)^※^
	Total	66	7.64 ± 1.82
			7.00 (3–12)^※^

(C) IED					
Age		*N*	Stages completed	*N*	EDS errors
6–7	(Boy)	34	7.88 ± 1.25	33	16.24 ± 10.33
			8.00 (3–9)^※^		16.00 (2–35)^※^
	(Girl)	20	8.10 ± 0.91	20	13.45 ± 11.52
			8.00 (7–9)^※^		11.00 (0–31)^※^
	Total	54	7.96 ± 1.13	53	15.19 ± 10.77
			8.00 (3–9)^※^		16.00 (0–35)^※^
8–9	(Boy)	36	8.31 ± 1.37	35	11.77 ± 10.22
			9.00 (2–9)^※^		8.00 (1–33)^※^
	(Girl)	32	8.06 ± 1.01	31	14.16 ± 10.53
			8.50 (6–9)^※^		10.00 (1–31)^※^
	Total	68	8.19 ± 1.21	66	12.89 ± 10.35
			9.00 (2–9)^※^		9.00 (1–33)^※^
10–12	(Boy)	44	8.50 ± 0.85	44	9.61 ± 10.19
			9.00 (7–9)^※^		5.00 (0–34)^※^
	(Girl)	23	8.78 ± 0.60	23	8.26 ± 9.20
			9.00 (7–9)^※^		5.00 (0–33)^※^
	Total	67	8.60 ± 0.78	67	9.15 ± 9.81
			9.00 (7–9)^※^		5.00 (0–34)^※^

(D) SST					
Age		*N*	Direction errors		Proportion of successful stops
6–7	(Boy)	35	10.31 ± 10.99		55.46 ± 12.95
			7.00 (0–43)^※^		
	(Girl)	25	3.80 ± 4.06	*	61.36 ± 11.47
			2.00 (0–13)^※^		
	Total	60	7.60 ± 9.32		57.92 ± 12.60
			4.00 (0–43)^※^		
8–9	(Boy)	43	6.58 ± 12.08		52.26 ± 11.40
			2.00 (0–71)^※^		
	(Girl)	37	2.05 ± 2.78		56.81 ± 9.62
			1.00 (0–11)^※^		
	Total	80	4.49 ± 9.29		54.36 ± 10.79
			1.00 (0–71)^※^		
10–12	(Boy)	45	3.73 ± 6.23		52.67 ± 9.31
			1.00 (0–29)^※^		
	(Girl)	26	2.23 ± 3.22		54.19 ± 9.03
			2.00 (0–14)^※^		
	Total	71	3.18 ± 5.35		53.23 ± 9.17
			1.00 (0–29)^※^		

※, Median (range).

### 3.1. Spatial working memory

The distributions of scores for the between errors in the four- and six-box problems are presented in Supplementary Figures S1A,B, respectively. The distribution pattern of the scores differed for each problem. On the four-box problem, the histogram showed a J-shaped distribution, whereby many participants made few errors, whereas some participants made some errors (Supplementary Figure S1A). Many participants, even in the 6–7 years group, showed few errors, and the number of participants who completed the stages with few errors increased with age (Supplementary Figure S1A). The ratio of the number of participants who completed the stages with zero errors accounted for 35.6, 44.7, and 75.0% in the 6–7, 8–9, and 10–12 years groups, respectively. Regarding the six-box problem, the histogram did not show a normal distribution (Supplementary Figure S1B). Compared with the four-box problem, the participants experienced increased errors as the task became increasingly difficult. The participants experienced fewer errors as age increased, but the errors were more moderate than those experienced in the four-box problem. The ratio of the number of participants who passed with zero errors accounted for 1.4, 0.0, and 14.5% in the 6–7, 8–9, and 10–12 years groups, respectively.

**Figure 2:**
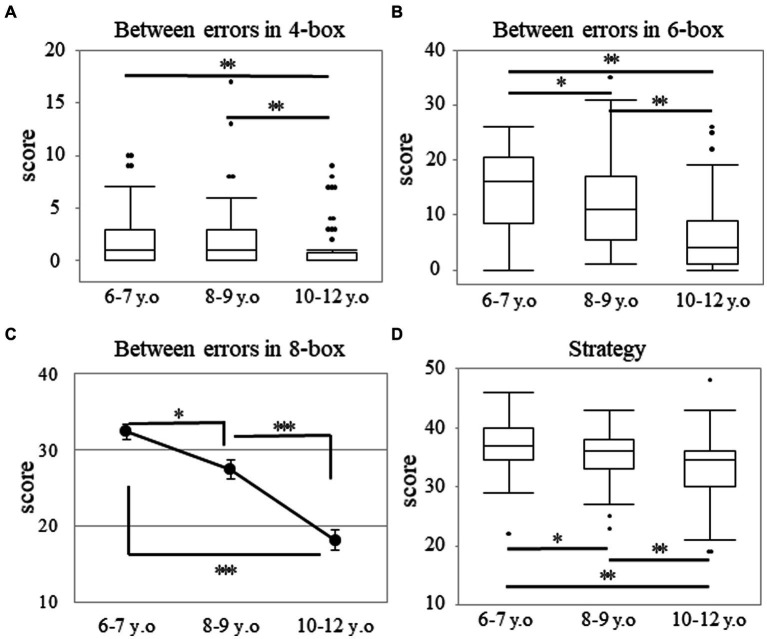
Between errors and strategy in the SWM task. Between errors in the four-box **(A)** and six-box **(B)** problems as well as strategy score **(D)** in the SWM task. The box plot represents the score for each age group **(A,B,D)**. *p < 0.017, **p < 0.003. Score of between errors in the eight-box problem **(C)** for the SWM task. Error bars indicate the standard error of the mean. *p < 0.05, ***p < 0.001. SWM, spatial working memory.

Through the Kruskal-Wallis test, a significant main effect of age was observed in the four- and six-box problems. In the four-box problem, the median (range) values were 1.00 (0–10), 1.00 (0–17), and 0.00 (0–9) in the 6–7, 8–9, and 10–12 years groups, respectively (*p* < 0.001; Figure 2A). In the six-box problem, those values were 16.00 (0–26), 11.00 (1–35), and 4.00 (0–26) in the 6–7, 8–9, and 10–12 years groups, respectively (*p* < 0.001; Figure 2B). *Post-hoc* analyses showed that the 10–12 years group made fewer errors than both the 6–7 (*p* < 0.003; *r* = 0.38) and 8–9 years groups (*p* < 0.003; *r* = 0.29). However, the values in the 6–7 and 8–9 years groups with regard to the four-box problem were not statistically significant (*p* = 0.200; Figure 2A). Regarding the six-box problem, there were differences among all the age groups (*p* < 0.017; *r* > 0.20; Figure 2B).

The Mann–Whitney U test did not show any statistically significant sex differences in each age group. In the four-box problem, the median (range) values were 2.00 (0–10) and 0.00 (0–10) among boys and girls in the 6–7 years group (*p* = 0.061). These values were 2.00 (0–8) and 0.00 (0–17) among boys and girls in the 8–9 years group (*p* = 0.063), and they were 0.00 (0–8) and 0.00 (0–9) among boys and girls in the 10–12 years group (*p* = 0.210). In the six-box problem, these values were 15.50 (0–26) and 16.00 (2–26) among boys and girls in the 6–7 years group (*p* = 0.592), 13.00 (1–26) and 8.00 (1–35) among boys and girls in the 8–9 years group (*p* = 0.124). These values were 4.00 (0–22) and 5.00 (0–26) among boys and girls in the 10–12 years group (*p* = 0.371).

The score of the between errors in the eight-box problem showed a normal distribution (*p* = 0.074), but the score of strategy did not (*p* < 0.001). In the eight-box problem, two-way analysis of variance revealed a significant main effect of age. The mean ± SD values were 32.34 ± 8.33, 27.41 ± 12.01, and 18.08 ± 11.48 in the 6–7, 8–9, and 10–12 years groups, respectively [*F* (2, 228) = 29.92; *p* < 0.001; *η_p_^2^* = 0.21]. *Post-hoc* analyses showed that there were differences among all the age groups (*p* < 0.05; Figure 2C). A statistically significant sex difference for each age group in the eight-box problem was not obtained through the independent *t*-test. The mean ± SD values were 32.33 ± 8.00 and 32.35 ± 8.89 among boys and girls in the 6–7 years group (*p* = 0.991), 28.56 ± 11.48 and 26.13 ± 12.59 among boys and girls in the 8–9 years group (*p* = 0.355), and 16.92 ± 12.10 and 20.07 ± 10.24 among boys and girls in the 10–12 years group (*p* = 0.250).

Regarding the strategy, there was a significant main effect of age. The median (range) values were 37.00 (22–46), 36.00 (23–43), and 34.50 (19–48) in the 6–7, 8–9, and 10–12 years groups, respectively (*p* < 0.001; Figure 2D), thereby indicating that there were differences among all the age groups (*p* < 0.017; *r* > 0.19). There were no statistically significant sex differences in each of the age groups. The median (range) values were 37.00 (29–42) and 38.00 (22–46) among the boys and girls in the 6–7 years group (*p* = 0.466). These values were 36.00 (23–43) and 36.00 (25–43) among the boys and girls in the 8–9 years group (*p* = 0.681) and were 32.50 (19–40) and 35.00 (19–48) among the boys and girls in the 10–12 years group (*p* = 0.340).

### 3.2. Stockings of Cambridge

**Figure 3:**
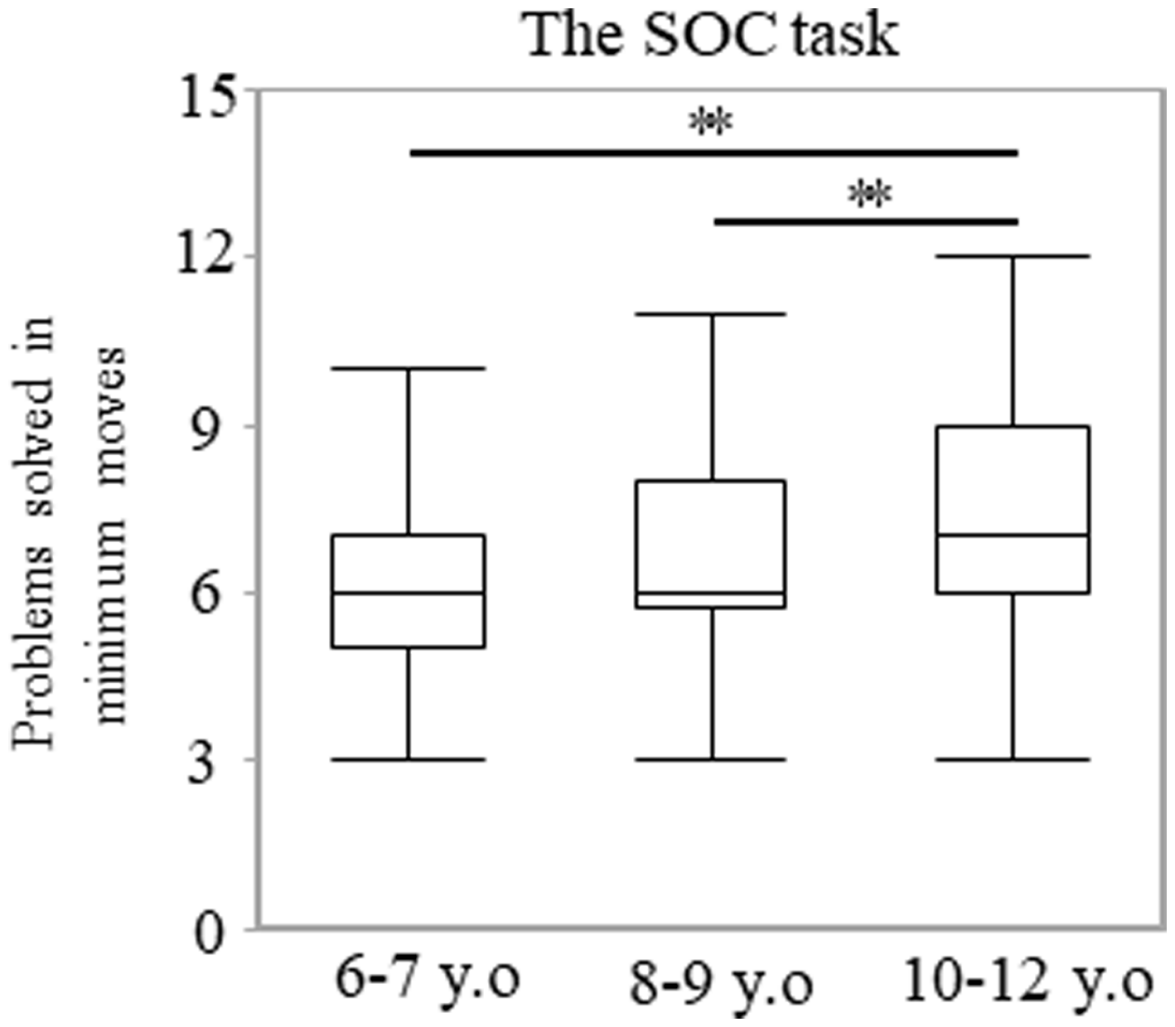
Problems solved in minimum moves in the SOC task. The box plot represents the score for each age group. **p < 0.003. SOC, Stockings of Cambridge.

The number of problems solved using the minimum moves possible did not show a normal distribution (*p* < 0.001). A significant main effect of age was observed. The median (range) values were 6.00 (3–10), 6.00 (3–11), and 7.00 (3–12) in the 6–7, 8–9, and 10–12 years groups, respectively (*p* < 0.001; Figure 3). *Post-hoc* analyses showed that the 10–12 years group solved more problems using the minimum moves possible than the 6–7 (*p* < 0.003; *r* = 0.38) or 8–9 years groups (*p* < 0.003; *r* = 0.29). However, there were no statistically significant differences between the 6–7 and 8–9 years groups (*p* = 0.246). There were no statistically significant differences in sex for each of the age groups. The median (range) values were 6.00 (3–10) and 7.00 (4–9) among the boys and girls in the 6–7 years group (*p* = 0.485). These values were 6.00 (3–11) and 6.50 (4–10) among the boys and girls in the 8–9 years group (*p* = 0.104), and they were 7.00 (3–12) and 8.00 (5–10) among the boys and girls in the 10–12 years group (*p* = 0.915).

### 3.3. Intra/extradimensional set shift

**Figure 4:**
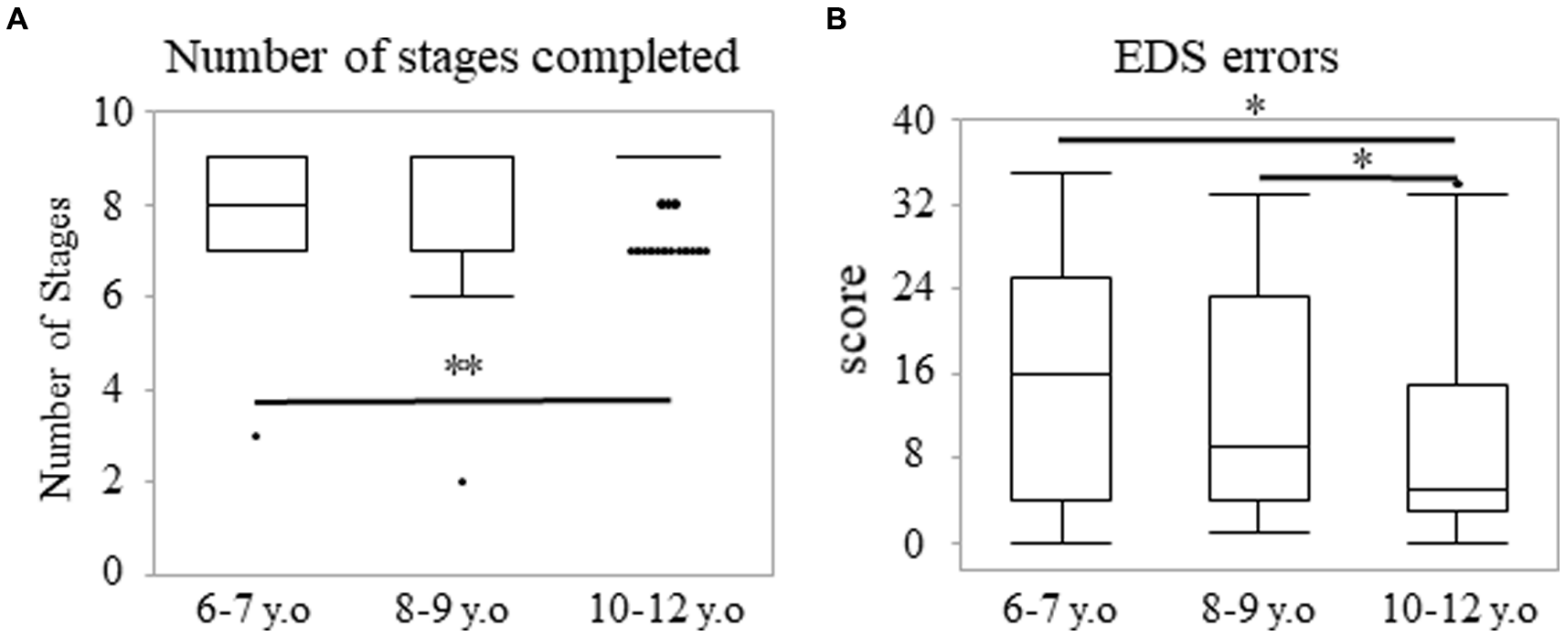
Number of stages completed and EDS errors in the IED task. Number of stages completed **(A)** and EDS errors **(B)** in the IED task. The box plot presents the score for each age group **(A,B)**. *p < 0.017, **p < 0.003. IED, intra/extradimensional set shift.

The score of the stages completed did not show a normal distribution for each year group (Supplementary Figure S2A). Many participants, even the youngest, reached Stage 9 owing to the ease of the task (Supplementary Figure S2A). The number of participants who reached Stage 9 increased among the older groups. The ratio of the participants who achieved Stage 9 accounted for 42.6, 60.3, and 77.6% in the 6–7, 8–9, and 10–12 years groups, respectively (Supplementary Figure S2A). A significant main effect of age was observed. The median (range) values were 8.00 (3–9), 9.00 (2–9), and 9.00 (7–9) in the 6–7, 8–9, and 10–12 years groups, respectively (*p* < 0.01). *Post-hoc* analyses showed that the 10–12 years group reached higher stages than the 6–7 years group (*p* < 0.003; *r* = 0.34; Figure 4A). However, there were no statistically significant differences between the 6–7 and 8–9 years groups (*p* = 0.142) and between the 8–9 and 10–12 years groups (*p* = 0.026). Statistically significant sex differences in each age group were not observed. The median (range) values were 8.00 (3–9) and 8.00 (7–9) among the boys and girls in the 6–7 years group (*p* = 0.651). These values were 9.00 (2–9) and 8.50 (6–9) among the boys and girls in the 8–9 years group (*p* = 0.123), and they were 9.00 (7–9) and 9.00 (7–9) among the boys and girls in the 10–12 years group (*p* = 0.172).

Regarding the EDS errors, the data exhibited a bimodal distribution with increased errors, thereby reflecting the failure in finding role changes (Supplementary Figure S2B). Some participants in the 6–7 years group showed fewer errors (Supplementary Figure S2B). However, the number of participants with fewer errors increased in the 10–12 years group. The ratio of the scores under seven accounted for 37.7, 40.9, and 65.7% in the 6–7, 8–9, and 10–12 years groups, respectively (Supplementary Figure S2B). There was a significant main effect of age. The median (range) values were 16.00 (0–35), 9.00 (1–33), and 5.00 (0–34) in the 6–7, 8–9, and 10–12 years groups, respectively (*p* < 0.01; Figure 4B). This result indicated that participants in the 10–12 years group made fewer errors in the EDS stage, compared to the 6–7 (*p* < 0.017; *r* = 0.26) or 8–9 years groups (*p* < 0.017; *r* = 0.21). However, there were no statistically significant differences between the 6–7 and 8–9 years groups (*p* = 0.350). There were no statistically significant sex differences in each of the age groups. The median (range) values were 16.00 (2–35) and 11.00 (0–31) among the boys and girls in the 6–7 years group (*p* = 0.188). These values were 8.00 (1–33) and 10.00 (1–31) among the boys and girls in the 8–9 years group (*p* = 0.381), and they were 5.00 (0–34) and 5.00 (0–33) among the boys and girls in the 10–12 years group (*p* = 0.827).

### 3.4. Stop signal task

**Figure 5:**
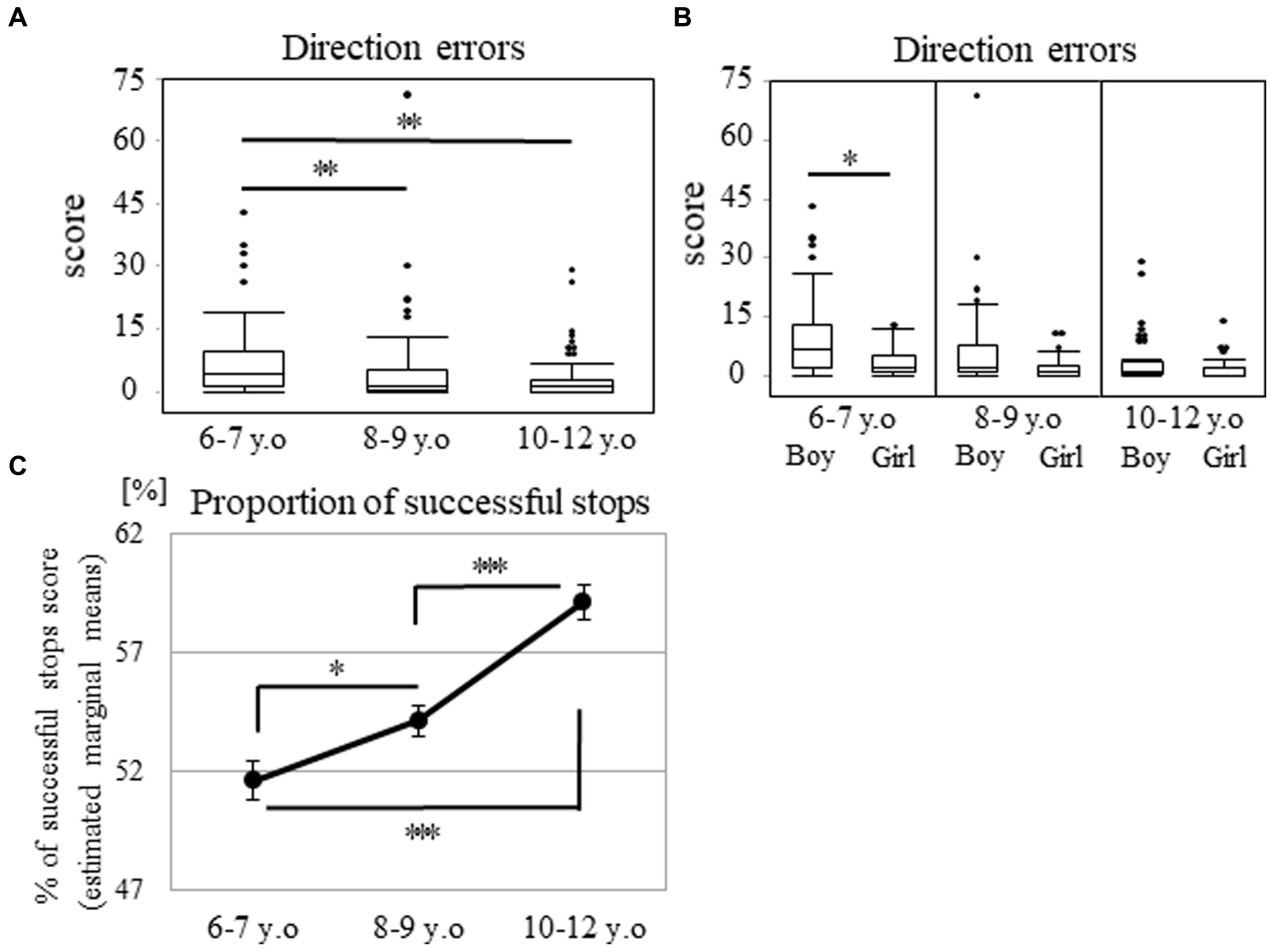
Direction errors and proportion of successful stops in the SST subtest. The box plot shows the score of direction errors for each age group **(A)** and that for the age and sex groups **(B)**. *p < 0.017, **p < 0.003. Proportion of successful stops for each age group **(C)**. Estimated marginal means represent the mean value for each category after adjusting the covariates. Error bars indicate the standard error of the mean. *p < 0.05, ***p < 0.001. SST, stop signal task.

Regarding the direction errors, the data exhibited a J-shaped distribution, whereby many participants made few errors, whereas some participants made some or many errors (Supplementary Figure S3). The number of participants with low errors increased in the older groups. The ratio of the errors under five accounted for 51.7, 75.0, and 83.1% in the 6–7, 8–9, and 10–12 years groups, respectively (Supplementary Figure S3). A significant main effect of age was observed. The median (range) values were 4.00 (0–43), 1.00 (0–71), and 1.00 (0–29) in the 6–7, 8–9, and 10–12 years groups, respectively (*p* < 0.001; Figure 5A). *Post-hoc* analyses showed that the 6–7 years group made more errors than the 8–9 (*p* < 0.003; *r* = 0.27) or 10–12 years groups (*p* < 0.003; *r* = 0.34). However, there were no statistically significant differences between the 8–9 and 10–12 years groups (*p* = 0.452). Additionally, there were significant sex differences in the 6–7 years group (Figure 5B). The median (range) values were 7.00 (0–43) and 2.00 (0–13) among the boys and girls in the 6–7 years group (*p* < 0.017; *r* = 0.34). These values were 2.00 (0–71) and 1.00 (0–11) among the boys and girls in the 8–9 years group (*p* = 0.037), and they were 1.00 (0–29) and 2.00 (0–14) among the boys and girls in the 10–12 years group (*p* = 0.396).

Regarding the proportion of successful stops, the score showed a normal distribution (*p* = 0.057). Two-way analysis of covariance, with the mean reaction time on the go trials as a covariate, demonstrated a main effect of age (57.92 ± 12.60, 54.36 ± 10.79, and 53.23 ± 9.17 in the 6–7, 8–9, and 10–12 years groups, respectively [*F* (2, 204) = 22.26; *p* < 0.001; *η_p_*^2^ = 0.18; Figure 5C]). This result indicated that the differences between all the age groups were significant (*p* < 0.05).

There were no statistically significant differences in sex for each of the age groups through one-way analysis of covariance [55.46 ± 12.95 and 61.36 ± 11.47 among the boys and girls in the 6–7 years group (*p* = 0.225), 52.26 ± 11.40 and 56.81 ± 9.62 among the boys and girls in the 8–9 years group (*p* = 0.290), and 52.67 ± 9.31 and 54.19 ± 9.03 among the boys and girls in the 10–12 years group (*p* = 0.488)].

### 3.5. Correlational analyses

Correlations among variables in the tasks are presented in Table 2. Between errors in the four-box problem were significantly correlated with between errors in the six- and eight-box problems as well as strategy for the SWM task (*r* = 0.455, 0.434, and 0.312, respectively) and the number of problems solved using the minimum moves possible in the SOC task (*r* = −0.318). Between errors in the six-box problem were significantly correlated with between errors in the eight-box problem and strategy for the SWM task (*r* = 0.719 and 0.702, respectively), the number of problems solved using the minimum moves possible in the SOC task (*r* = −0.345), and the direction errors in the SST subtest (*r* = 0.277). Between errors in the eight-box problem were significantly related to the strategy score in the SWM task (*r* = 0.693), the number of problems solved using the minimum moves possible in the SOC task (*r* = −0.364), and the direction errors in the SST subtest (*r* = 0.318). The strategy score was significantly correlated with the direction errors in the SST subtest (*r* = 0.285). In the IED task, the scores of the stages completed were significantly related to the EDS errors (*r* = −0.755). In the SST subtest, the direction errors were significantly correlated with the proportion of successful stops (*r* = −0.629).

**Table 2:** Correlation matrix for task variables.

	SWM				SOC	IED		SST	
	BE			ST	PS	SC	EDS	DE	PR
Variables	4-box	6-box	8-box						
**SWM**									
Between errors (4-box)	–	0.455^*^	0.434^*^	0.312^*^	−0.318^*^	−0.106	0.104	0.226	−0.080
(6-box)		–	0.719^*^	0.702^*^	−0.345^*^	−0.214	0.127	0.277^*^	−0.021
(8-box)			–	0.693^*^	−0.364^*^	−0.173	0.145	0.318^*^	−0.073
Strategy				–	−0.215	−0.204	0.170	0.285^*^	−0.032
**SOC**									
Problems solved in minimum moves					–	0.012	0.033	−0.118	−0.085
**IED**									
Stages completed						–	−0.755^*^	−0.160	0.011
EDS errors							–	0.145	0.002
**SST**									
Direction errors								–	−0.629^*^
Proportion of successful stops									–

* *p* < 0.001; SWM, spatial working memory; SOC, Stockings of Cambridge; IED, intra/ extradimensional set shift; SST, stop signal task; BE, between errors; ST, strategy score; PS, problems solved in minimum moves; SC, stages completed; EDS, EDS errors; DE, direction errors; PR, proportion of successful stops.

### 3.6. Comparison with previously reported normative values

**Figure 6:**
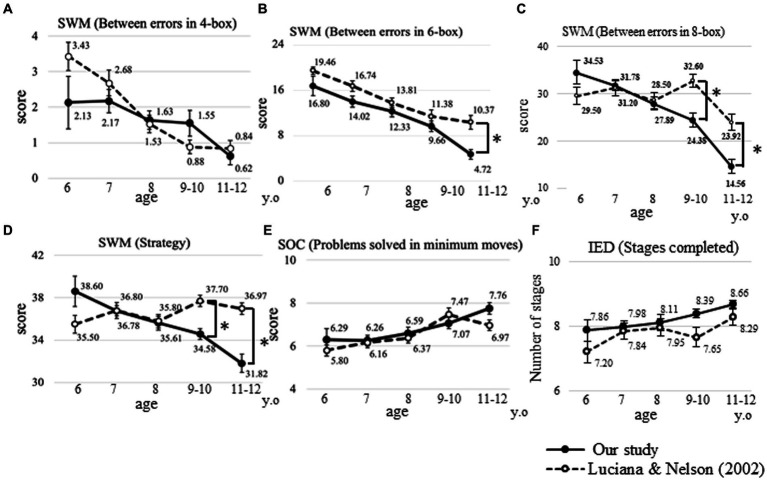
Comparison between previously reported normative values. Comparison of our data regarding between errors in the four-box **(A)**, six-box **(B)**, and eight-box problems **(C)**, strategy **(D)** in the SWM, problems solved using the minimum moves possible **(E)** in the SOC, and stages completed **(F)** in the IED task, compared to the normative values presented by Luciana and Nelson (2002). Error bars indicate the standard error of the mean. *p < 0.01. Solid circle: our study, open circle: modified from Luciana and Nelson (2002); SWM: spatial working memory; SOC: Stockings of Cambridge; IED: intra/extradimensional set shift.

Figure 6 shows a comparison of our data with the normative values presented by Luciana and Nelson (2002) for the SWM, SOC, and IED subtests.

Regarding the between errors in the four-box problem, at the age of 6 years, the score was lower in our data. However, there were no statistically significant differences between the previously reported data and our data. Regarding the between errors in the six-box problem, the slope of the score was almost similar, but it was significantly lower at 11–12 years in our data (4.7 ± 5.2) compared with previously reported data (10.4 ± 7.3, *p* < 0.001). In the eight-box problem, the scores were significantly lower at 9–10 and 11–12 years in our data (24.4 ± 13.8 and 14.6 ± 9.1) compared with previously reported data (32.6 ± 9.4 and 23.9 ± 10.0; *p* < 0.001, respectively). Regarding the score of the strategy in the SWM task, the previously reported data did not show obvious differences between ages, and the score was also significantly lower at 9–10 and 11–12 years in our data (34.6 ± 4.4 and 31.8 ± 5.4) compared to previously reported data (37.7 ± 3.5 and 37.0 ± 3.2; *p* < 0.001, respectively).

In the SOC and IED tasks, no statistically significant differences were observed between our data and previously reported data (Figure 6).

## 4. Discussion

The SWM task aimed to evaluate spatial working memory and heuristic strategies. This study shows significant differences between all the age groups in the between errors for the six- and eight-box problems, thereby suggesting that spatial working memory develops at a younger age.

Spatial working memory performance depends on the interaction between the ability required for each problem and developmental trends. As previously reported (Luciana and Nelson, 1998), there were no statistically significant differences between the 6–7 and 8–9 years groups in the four-box problem, and this result indicated that children in lower grades might have reached the saturation value on the four-box problem. According to a previous neuropsychological study, the performance of patients with injuries to the right frontal lobe was not significantly impaired compared to that of patients without injuries to the right frontal lobe and that of controls in the four-box problem. However, their performance was significantly impaired in the increasingly difficult problems (Chase et al., 2008). Contrary to the four-box problem, the six-box problem showed significant differences between all the age groups. However, in this study, both the four-and six-box problems did not appear in the normal distribution. Therefore, the eight-box problem may have a more sensitive structure than the four- or six-box problems for estimating SWM among elementary school children in Japan.

The SOC task was also a spatial planning test. As shown in Figure 3, significant differences in minimum moves were found between the 8–9 and 10–12 years groups, rather than between the 6–7 and 8–9 years groups. This result indicates that the ability to plan develops after 8 years of age.

In the IED task, the ratio of the participants who reached Stage 9 was very high. Additionally, our data on the number of stages completed showed results similar to those reported in previous studies (Luciana and Nelson, 2002; De Luca et al., 2003; Green et al., 2019). Owing to the ceiling effect, this outcome measure can be useful in detecting severe dysfunctions associated with the ability to understand rules, but it may not be a sensitive tool for estimating developmental trends and individual differences among school-aged children. However, the EDS errors, which estimate the flexibility of attention, were significantly different between the 8–9 and 10–12 years groups, rather than between the 6–7 and 8–9 years groups. These findings indicate that cognitive flexibility develops after 8 years of age, and the EDS errors may be more useful for elementary school-aged children.

In the SST subtest, the direction errors and the proportion of successful stops showed significant differences between the 6–7 and 8–9 years groups, which may represent inhibition improvement at a younger age. The number of direction errors in the SST subtest was lower among girls, compared to boys in the 6–7 years group. This may reflect differences in functional developmental trajectories between males and females. It was also reported that typically developing four-year-old girls exhibit more efficient brain functioning than boys in an inhibitory task (Cuevas et al., 2016). Direction errors are considered to reflect deficits in sustained attention and inhibition, both of which are mainly handled by the frontal and parietal cortices (Bari and Robbins, 2013; Langner and Eickhoff, 2013). Findings in structural magnetic resonance imaging also indicate that peak gray matter volumes in the frontal and parietal cortices occur 1–2 years earlier among females (Lenroot et al., 2007), and females reach their fractional anisotropy plateaus at an earlier age in the bilateral superior longitudinal fasciculus compared to males (Chen Z. et al., 2016). Therefore, increasingly successful performance in the inhibitory task among girls aged 6–7 years may be associated with earlier structural maturation among females.

Regarding correlational analysis, in addition to significant correlations between the variables within similar tasks, the SWM variables showed weak but significant correlations between the SOC index and direction errors in the SST subtest (Table 2). The former correlations may reflect that the SOC task assesses not only planning but also spatial working memory, and the latter may represent the aspect that working memory and inhibitory control support each other and co-occur (Diamond, 2013). However, the variables in the IED task were not significantly correlated with the variables in the other tasks. One possible explanation for this finding is that flexibility evaluated by the IED task might be an independent domain among other subcomponents of EFs. The other possible explanation is that these outcome measures may merely focus more on the aspect of higher-order functions to understand rules. Further studies are required to clarify this explanation.

This study presents different developmental trajectories depending on components of EFs summarized in Table 3, which may be organized hierarchically. In other words, higher-order EFs, such as planning and flexibility might be built on more basic functions, such as spatial working memory and inhibition. For successful performance in planning and flexibility, it is necessary to maintain information and inhibit impulsive responses. This hierarchy is consistent with Diamond’s framework of EFs, comprehensively reviewed in terms of developmental and organizational perspectives (Diamond, 2013).

**Table 3:** Summary of results for age differences.

Trajectory	CANTAB
6–7 << 8–9 = 10–12	Direction errors (SST)
6–7 = 8–9 << 10–12	Between errors in the 4-box problem (SWM)
6–7 < 8–9 < 10–12	Between errors in the 6-box problem (SWM)
	Between errors in the 8-box problem (SWM)
	Strategy (SWM)
	Proportion of successful stops (SST)
6–7 < 10–12	Stages completed (IED)
6–7 = 8–9 < 10–12	Problems solved in minimum moves (SOC)
	EDS errors (IED)

SWM, spatial working memory; SOC, Stockings of Cambridge; IED, intra/extradimensional set shift; SST, stop signal task.

The hierarchy of EFs may also reflect different anatomical maturation trajectories in the subregions within the frontal cortex. According to the findings of previous studies involving adults, the SWM task is significantly related to the right pars opercularis (BA44, Chase et al., 2008). Additionally, a neuroimaging study on the SWM tasks among children indicated that improved performance was associated with cortical thickness in the right inferior frontal gyrus (Zhong et al., 2014). In the SST subtest, the findings of structural magnetic resonance imaging involving children showed associations between improved response inhibition performance and increased level of fractional anisotropy in white matter microstructure within the right pars opercularis (Madsen et al., 2010) and a relatively larger cortical surface area of the right pars opercularis (Curley et al., 2018). However, during the SOC task, more broad brain areas, including the left mid-dorsolateral PFC (BA9, Owen et al., 1996), the left dorsolateral PFC (BA9/46), and the left orbitofrontal cortex (BA 14, Beauchamp et al., 2003) were activated in positron emission tomography studies involving adults. Similarly, the IED task has been reported to be associated with the left anterior PFC and the right dorsolateral PFC (BA 10 and 9/46, Rogers et al., 2000). The maturational sequence within the frontal lobe progresses back-to-front from the precentral gyrus to the dorsolateral PFC (Gogtay et al., 2004). According to these and previous findings, the functional developmental trajectories from the ventrolateral PFC, which is responsible for more basic EFs, to the dorsolateral PFC, which is involved in higher-order functions, might be parallel to the structural sequence of maturation within the frontal cortex. The higher hierarchy functions mobilize the left hemisphere as well as the right hemisphere. This may also require the maturation of interhemispheric connections. Owing to the tasks involving different complex rules used in this study, the hierarchy of functions might be explained by the hypothesis that the development of rule use may be associated with developmental changes in its neural bases during childhood (Bunge and Zelazo, 2006).

Comparing our data with the normative values presented by Luciana and Nelson (2002), there were no significant differences in the higher hierarchized EFs, such as planning, based on the scores of the problems solved using the minimum moves possible in the SOC task and flexibility based on the scores of the stages completed in the IED task. However, there were significant differences in the between errors for the six- and eight-box problems as well as the strategy scores in the SWM task at an older age. This might indicate a different developmental trajectory between Japanese and American children. This further indicates that standard values are required for each ethnic group. Contrarily, a previous study reported a positive relationship between computer use and EFs skills among 5–12-year-old children (Rosenqvist et al., 2016). This may have resulted from the degree to which the children were exposed to computers in their daily lives. There is a nearly 15-year gap from the time between the previous study and this study, during which computers have become increasingly familiar and fundamental in the environments surrounding children. Furthermore, older children use computers more than younger children (Rosenqvist et al., 2016). Because this study did not control for some confounding variables, including the frequency of computer use, we may obtain a better understanding by making them match among studies.

The impairment of EFs has been demonstrated in various neurodevelopmental disorders, such as autism spectrum disorders and attention deficit hyperactivity disorder (Corbett et al., 2009; Yerys et al., 2009; Nagatani et al., 2012; Coghill et al., 2014; Chen S. F. et al., 2016). Over the recent years, promising interventions, including cognitive remediation and non-invasive brain stimulation methods for executive dysfunction, have been developed (Dandil et al., 2020; Khaleghi et al., 2020). In Japan, there are no normative values of some components of EFs evaluated using CANTAB. The findings of this study will enable the assessment of some aspects of EFs to understand patients’ needs for appropriate interventions.

In conclusion, this study demonstrates that the performance of all CANTAB subtests changed with age during elementary school. In each of the subtests, the performances in the SWM and SST developed at a younger age, whereas those in the SOC and IED tasks developed at an older age. Additionally, in the SST subtest, girls made fewer direction errors than boys did in the 6–7 years group. To the best of our knowledge, this study is the first to present normative data of four CANTAB subtests according to age and sex among school-aged children in Japan. This study also indicates that it was necessary to refer to appropriate normative values for the participants’ demographics, such as age and sex, to evaluate their EFs. We expect that the findings of this study will be used to develop effective tools for the early detection of and support for children with executive dysfunction.

This study has some potential limitations. First, we did not examine test–retest reliabilities. Further evaluation of the data’s reliability should be addressed in future studies. Moreover, future studies establishing the validity using a criterion measure such as BRIEF (Gioia et al., 2000) will provide more robust psychometric properties of the CANTAB for school-aged children.

Second, this study lacks a longitudinal design. Future studies using a longitudinal design would enable an increasingly reliable examination of the changes in each component of EFs throughout children’s development.

Third, this study does not include confounding factors. For example, intelligence, socioeconomic status, and frequency of computer use have been shown to be associated with EFs (Roca et al., 2010; Rosenqvist et al., 2016; Last et al., 2018). Future studies should consider these variables to ascertain purer age-related changes in EFs.

## Data availability statement

The datasets presented in this article are not readily available because it is restricted by Institutional Review Board of Osaka University Hospital. Requests to access the datasets should be directed to the corresponding author.

## Ethics statement

The studies involving human participants were reviewed and approved by the Institutional Review Board of Osaka University Hospital. Written informed consent to participate in this study was provided by the participants’ legal guardian/next of kin.

## Author contributions

SA: data collection, data analysis, and manuscript writing. FN: data collection. KK-S: study design and data collection. YO: data analysis. MT: data analysis and manuscript writing. IM: study design, data analysis, and manuscript writing. All authors contributed to the article and approved the submitted version.

## Funding

This study was supported in part by research grants from the Ministry of Education, Culture, Sports, Science and Technology of Japan (15K12721 and 6H03273 to MT).

## Conflict of interest

The authors declare that the research was conducted in the absence of any commercial or financial relationships that could be construed as a potential conflict of interest.

## Publisher’s note

All claims expressed in this article are solely those of the authors and do not necessarily represent those of their affiliated organizations, or those of the publisher, the editors and the reviewers. Any product that may be evaluated in this article, or claim that may be made by its manufacturer, is not guaranteed or endorsed by the publisher.

## Footnotes

^1^ https://www.cambridgecognition.com/cantab/

## References

[ref1] Anderson P. (2002). Assessment and development of executive function (EF) during childhood. Child Neuropsychol. 8, 71–82. doi: 10.1076/chin.8.2.71.8724 12638061

[ref2] Baddeley A. (1992). Working memory. Science 255, 556–559. doi: 10.1126/science.1736359 1736359

[ref3] Bari A. Robbins T. W. (2013). Inhibition and impulsivity: behavioral and neural basis of response control. Prog. Neurobiol. 108, 44–79. doi: 10.1016/j.pneurobio.2013.06.005 23856628

[ref4] Bathelt J. Holmes J. Astle D. E. Centre for Attention Learning and Memory (CALM) Team (2018). Data-driven subtyping of executive function-related behavioral problems in children. J. Am. Acad. Child Adolesc. Psychiatry 57, 252–262.e4. doi: 10.1016/j.jaac.2018.01.014, PMID: 29588051 PMC5889789

[ref5] Beauchamp M. H. Dagher A. Aston J. A. D. Doyon J. (2003). Dynamic functional changes associated with cognitive skill learning of an adapted version of the tower of London task. NeuroImage 20, 1649–1660. doi: 10.1016/j.neuroimage.2003.07.003, PMID: 14642475

[ref6] Best J. R. Miller P. H. (2010). A developmental perspective on executive function. Child Dev. 81, 1641–1660. doi: 10.1111/j.1467-8624.2010.01499.x, PMID: 21077853 PMC3058827

[ref7] Best J. R. Miller P. H. Jones L. L. (2009). Executive functions after age 5: changes and correlates. Dev. Rev. 29, 180–200. doi: 10.1016/j.dr.2009.05.002, PMID: 20161467 PMC2792574

[ref8] Borella E. Carretti B. Pelegrina S. (2010). The specific role of inhibition in reading comprehension in good and poor comprehenders. J. Learn. Disabil. 43, 541–552. doi: 10.1177/0022219410371676, PMID: 20606207

[ref9] Bunge S. A. Zelazo P. D. (2006). A brain-based account of the development of rule use in childhood. Curr. Dir. Psychol. Sci. 15, 118–121. doi: 10.1111/j.0963-7214.2006.00419.x

[ref10] Chase H. W. Clark L. Sahakian B. J. Bullmore E. T. Robbins T. W. (2008). Dissociable roles of prefrontal subregions in self-ordered working memory performance. Neuropsychologia 46, 2650–2661. doi: 10.1016/j.neuropsychologia.2008.04.021, PMID: 18556028

[ref11] Chen S. F. Chien Y. L. Wu C. T. Shang C. Y. Wu Y. Y. Gau S. S. (2016). Deficits in executive functions among youths with autism spectrum disorders: an age-stratified analysis. Psychol. Med. 46, 1625–1638. doi: 10.1017/S0033291715002238, PMID: 26997535 PMC4873936

[ref12] Chen Z. Zhang H. Yushkevich P. A. Liu M. Beaulieu C. (2016). Maturation along white matter tracts in human brain using a diffusion tensor surface model tract-specific analysis. Front. Neuroanat. 10:9. doi: 10.3389/fnana.2016.00009, PMID: 26909027 PMC4754466

[ref13] Cho I. Hosseini-Kamkar N. Song H. Morton J. B. (2023). Culture, executive functions, and academic achievement. Front. Psychol. 14:1100537. doi: 10.3389/fpsyg.2023.1100537, PMID: 37251073 PMC10214865

[ref14] Coghill D. R. Hayward D. Rhodes S. M. Grimmer C. Matthews K. (2014). A longitudinal examination of neuropsychological and clinical functioning in boys with attention deficit hyperactivity disorder (ADHD): improvements in executive functioning do not explain clinical improvement. Psychol. Med. 44, 1087–1099. doi: 10.1017/S0033291713001761, PMID: 23866120

[ref15] Corbett B. A. Constantine L. J. Hendren R. Rocke D. Ozonoff S. (2009). Examining executive functioning in children with autism spectrum disorder, attention deficit hyperactivity disorder and typical development. Psychiatry Res. 166, 210–222. doi: 10.1016/j.psychres.2008.02.005, PMID: 19285351 PMC2683039

[ref16] Cuevas K. Calkins S. D. Bell M. A. (2016). To stroop or not to stroop: sex-related differences in brain-behavior associations during early childhood. Psychophysiology 53, 30–40. doi: 10.1111/psyp.12464 26681615 PMC4685738

[ref17] Curley L. B. Newman E. Thompson W. K. Brown T. T. Hagler D. J. Akshoomoff N.. (2018). Cortical morphology of the pars opercularis and its relationship to motor-inhibitory performance in a longitudinal, developing cohort. Brain Struct. Funct. 223, 211–220. doi: 10.1007/s00429-017-1480-5, PMID: 28756486 PMC5772141

[ref18] Dajani D. R. Uddin L. Q. (2015). Demystifying cognitive flexibility: implications for clinical and developmental neuroscience. Trends Neurosci. 38, 571–578. doi: 10.1016/j.tins.2015.07.003, PMID: 26343956 PMC5414037

[ref19] Dandil Y. Smith K. Kinnaird E. Toloza C. Tchanturia K. (2020). Cognitive remediation interventions in autism spectrum condition: a systematic review. Front. Psych. 11:722. doi: 10.3389/fpsyt.2020.00722, PMID: 32793009 PMC7393993

[ref20] De Luca C. R. Wood S. J. Anderson V. Buchanan J. A. Proffitt T. M. Mahony K.. (2003). Normative data from the Cantab. I: development of executive function over the lifespan. J. Clin. Exp. Neuropsychol. 25, 242–254. doi: 10.1076/jcen.25.2.242.13639, PMID: 12754681

[ref21] Diamond A. (2013). Executive functions. Annu. Rev. Psychol. 64, 135–168. doi: 10.1146/annurev-psych-113011-143750 23020641 PMC4084861

[ref22] Fuster J. M. (2002). Frontal lobe and cognitive development. J. Neurocytol. 31, 373–385. doi: 10.1023/A:1024190429920 12815254

[ref23] Gathercole S. E. Pickering S. J. Knight C. Stegmann Z. (2004). Working memory skills and educational attainment: evidence from national curriculum assessments at 7 and 14 years of age. Appl. Cogn. Psychol. 18, 1–16. doi: 10.1002/acp.934

[ref24] Gioia G. A. Isquith P. K. Guy S. C. Kenworthy L. (2000). Test review behavior rating inventory of executive function. Child Neuropsychol. 6, 235–238. doi: 10.1076/chin.6.3.235.3152 11419452

[ref25] Gogtay N. Giedd J. N. Lusk L. Hayashi K. M. Greenstein D. Vaituzis A. C.. (2004). Dynamic mapping of human cortical development during childhood through early adulthood. Proc. Natl. Acad. Sci. U. S. A. 101, 8174–8179. doi: 10.1073/pnas.0402680101, PMID: 15148381 PMC419576

[ref26] Green R. Till C. Al-Hakeem H. Cribbie R. Téllez-Rojo M. M. Osorio E.. (2019). Assessment of neuropsychological performance in Mexico City youth using the Cambridge neuropsychological test automated battery (CANTAB). J. Clin. Exp. Neuropsychol. 41, 246–256. doi: 10.1080/13803395.2018.1529229, PMID: 30336715 PMC6910779

[ref27] Khaleghi A. Zarafshan H. Vand S. R. Mohammadi M. R. (2020). Effects of non-invasive neurostimulation on autism spectrum disorder: a systematic review. Clin. Psychopharmacol. Neurosci. 18, 527–552. doi: 10.9758/cpn.2020.18.4.527, PMID: 33124586 PMC7609207

[ref28] Langner R. Eickhoff S. B. (2013). Sustaining attention to simple tasks: a meta-analytic review of the neural mechanisms of vigilant attention. Psychol. Bull. 139, 870–900. doi: 10.1037/a0030694, PMID: 23163491 PMC3627747

[ref29] Last B. S. Lawson G. M. Breiner K. Steinberg L. Farah M. J. (2018). Childhood socioeconomic status and executive function in childhood and beyond. PLoS One 13:e0202964. doi: 10.1371/journal.pone.0202964, PMID: 30142188 PMC6108482

[ref30] Lenroot R. K. Gogtay N. Greenstein D. K. Wells E. M. Wallace G. L. Clasen L. S.. (2007). Sexual dimorphism of brain developmental trajectories during childhood and adolescence. NeuroImage 36, 1065–1073. doi: 10.1016/j.neuroimage.2007.03.053 17513132 PMC2040300

[ref31] Luciana M. (2003). Practitioner review: computerized assessment of neuropsychological function in children: clinical and research applications of the Cambridge neuropsychological testing automated battery (CANTAB). J. Child. Psychol. Psychi. 44, 649–663. doi: 10.1111/1469-7610.00152, PMID: 12831110

[ref32] Luciana M. Nelson C. A. (1998). The functional emergence of prefrontally-guided working memory systems in four- to eight-year-old children. Neuropsychologia 36, 273–293. doi: 10.1016/S0028-3932(97)00109-7, PMID: 9622192

[ref33] Luciana M. Nelson C. A. (2002). Assessment of neuropsychological function through use of the Cambridge neuropsychological testing automated battery: performance in 4- to 12-year-old children. Dev. Neuropsychol. 22, 595–624. doi: 10.1207/S15326942DN2203_3 12661972

[ref34] MacLeod C. M. (2007). “The concept of inhibition in cognition” in Inhibition in cognition. eds. Gorfein D. S. MacLeod C. M. (Washington, DC: American Psychological Association), 3–23.

[ref35] Madsen K. S. Baaré W. F. C. Vestergaard M. Skimminge A. Ejersbo L. R. Ramsøy T. Z.. (2010). Response inhibition is associated with white matter microstructure in children. Neuropsychologia 48, 854–862. doi: 10.1016/j.neuropsychologia.2009.11.001, PMID: 19909763

[ref36] Nagatani F. Matsuzaki J. Eto M. Kagitani-Shimono K. Mohri I. Taniike M. (2012). Assessment of executive function using the behavior rating inventory of executive function (BRIEF) and the Cambridge neuropsychological test automated battery (CANTAB) in young children with attention deficit/hyperactivity disorder, inattention type. J. Brain Sci. 39, 5–21. doi: 10.20821/jbs.39.0_5

[ref37] Nitschke K. Köstering L. Finkel L. Weiller C. Kaller C. P. (2017). A meta-analysis on the neural basis of planning: activation likelihood estimation of functional brain imaging results in the tower of London task. Hum. Brain Mapp. 38, 396–413. doi: 10.1002/hbm.23368, PMID: 27627877 PMC6867129

[ref38] Owen A. M. Downes J. J. Sahakian B. J. Polkey C. E. Robbins T. W. (1990). Planning and spatial working memory following frontal lobe lesions in man. Neuropsychologia 28, 1021–1034. doi: 10.1016/0028-3932(90)90137-D, PMID: 2267054

[ref39] Owen A. M. Doyon J. Petrides M. Evans A. C. (1996). Planning and spatial working memory: a positron emission tomography study in humans. Eur. J. Neurosci. 8, 353–364. doi: 10.1111/j.1460-9568.1996.tb01219.x, PMID: 8714706

[ref40] Owen A. M. Roberts A. C. Polkey C. E. Sahakian B. J. Robbins T. W. (1991). Extra-dimensional versus intra-dimensional set shifting performance following frontal lobe excisions, temporal lobe excisions or amygdalo-hippocampectomy in man. Neuropsychologia 29, 993–1006. doi: 10.1016/0028-3932(91)90063-E, PMID: 1762678

[ref41] Robbins T. W. James M. Owen A. M. Sahakian B. J. Lawrence A. D. McInnes L.. (1998). A study of performance on tests from the CANTAB battery sensitive to frontal lobe dysfunction in a large sample of normal volunteers: implications for theories of executive functioning and cognitive aging. J. Int. Neuropsychol. Soc. 4, 474–490. doi: 10.1017/S1355617798455073, PMID: 9745237

[ref42] Robbins T. W. James M. Owen A. M. Sahakian B. J. McInnes L. Rabbitt P. (1994). Cambridge neuropsychological test automated battery (CANTAB): a factor analytic study of a large sample of normal elderly volunteers. Dementia 5, 266–281. PMID: 7951684 10.1159/000106735

[ref43] Roca M. Parr A. Thompson R. Woolgar A. Torralva T. Antoun N.. (2010). Executive function and fluid intelligence after frontal lobe lesions. Brain 133, 234–247. doi: 10.1093/brain/awp269, PMID: 19903732 PMC2801324

[ref44] Rogers R. D. Andrews T. C. Grasby P. M. Brooks D. J. Robbins T. W. (2000). Contrasting cortical and subcortical activations produced by attentional-set shifting and reversal learning in humans. J. Cogn. Neurosci. 12, 142–162. doi: 10.1162/089892900561931, PMID: 10769312

[ref45] Romine C. B. Reynolds C. R. (2005). A model of the development of frontal lobe functioning: findings from a meta-analysis. Appl. Neuropsychol. 12, 190–201. doi: 10.1207/s15324826an1204_2 16422660

[ref46] Roque D. T. Teixeira R. A. A. Zachi E. C. Ventura D. F. (2011). The use of the Cambridge neuropsychological test automated battery (CANTAB) in neuropsychological assessment: application in Brazilian research with control children and adults with neurological disorders. Psychol. Neurosci. 4, 255–265. doi: 10.3922/j.psns.2011.2.011

[ref47] Rosenqvist J. Lahti-Nuuttila P. Holdnack J. Kemp S. L. Laasonen M. (2016). Relationship of TV watching, computer use, and reading to children’s neurocognitive functions. J. Appl. Dev. Psychol. 46, 11–21. doi: 10.1016/j.appdev.2016.04.006

[ref48] Yerys B. E. Wallace G. L. Harrison B. Celano M. J. Giedd J. N. Kenworthy L. E. (2009). Set-shifting in children with autism spectrum disorders: reversal shifting deficits on the Intradimensional/extradimensional shift test correlate with repetitive behaviors. Autism 13, 523–538. doi: 10.1177/1362361309335716, PMID: 19759065 PMC3018342

[ref49] Yu Q. Abe N. King A. Yoon C. Liberzon I. Kitayama S. (2019). Cultural variation in the gray matter volume of the prefrontal cortex is moderated by the dopamine D4 receptor gene (DRD4). Cereb. Cortex 29, 3922–3931. doi: 10.1093/cercor/bhy271, PMID: 30364935

[ref50] Zhong J. Rifkin-Graboi A. Ta A. T. Yap K. L. Chuang K. H. Meaney M. J.. (2014). Functional networks in parallel with cortical development associate with executive functions in children. Cereb. Cortex 24, 1937–1947. doi: 10.1093/cercor/bht051, PMID: 23448875

[ref51] Zorza J. P. Marino J. Mesas A. A. (2016). Executive functions as predictors of school performance and social relationships: primary and secondary school students. Span. J. Psychol. 19:e23. doi: 10.1017/sjp.2016.23 27169746

