# Supplementary figures and images for: Examining normative values using the Cambridge neuropsychological test automated battery and developmental traits of executive functions among elementary school-aged children in Japan
Source: Front Psychol. 2023 Aug 16;14:1141628. doi: 10.3389/fpsyg.2023.1141628 (PMC10469330; doi:10.3389/fpsyg.2023.1141628)

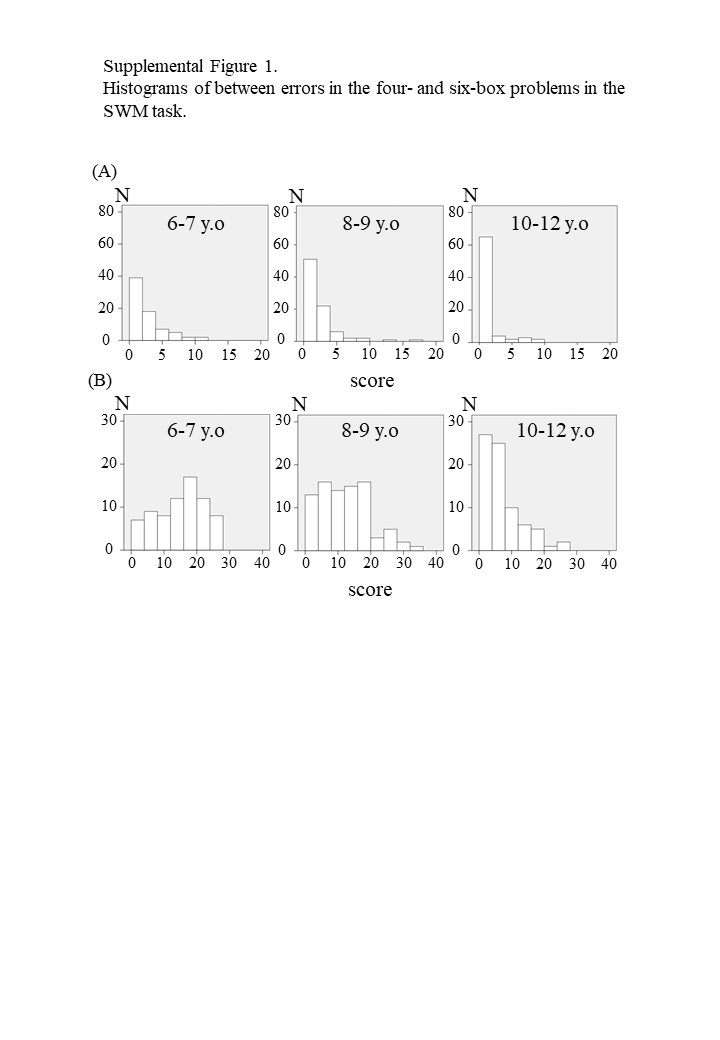

Supplement: Supplementary file 1 [file Image_1.TIF]

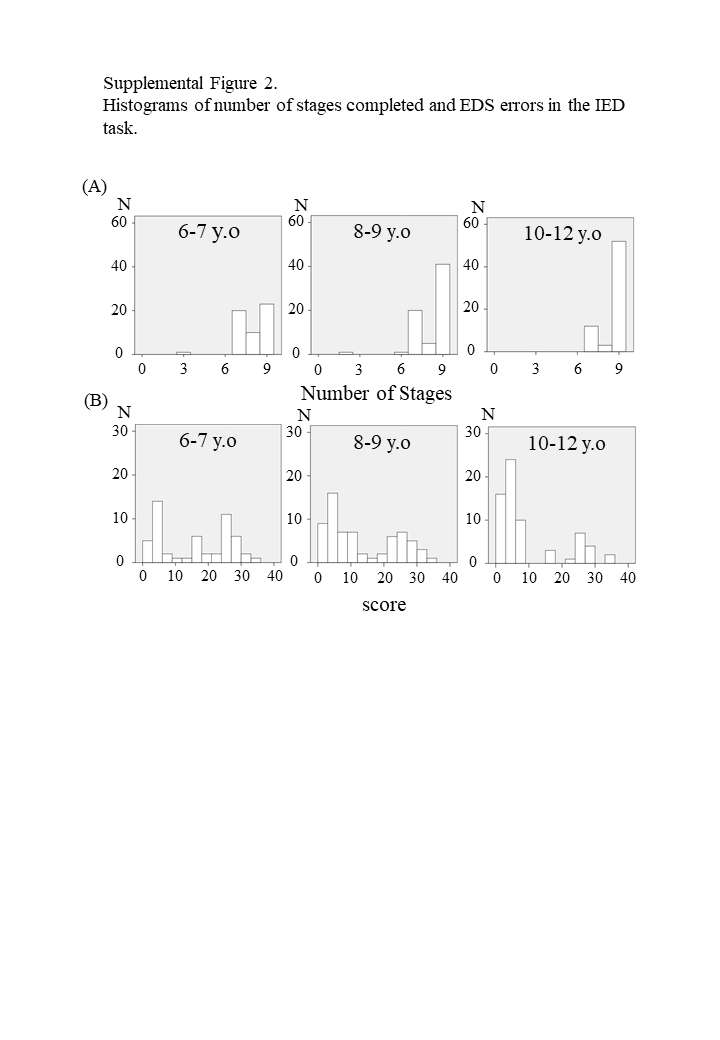

Supplement: Supplementary file 2 [file Image_2.TIF]

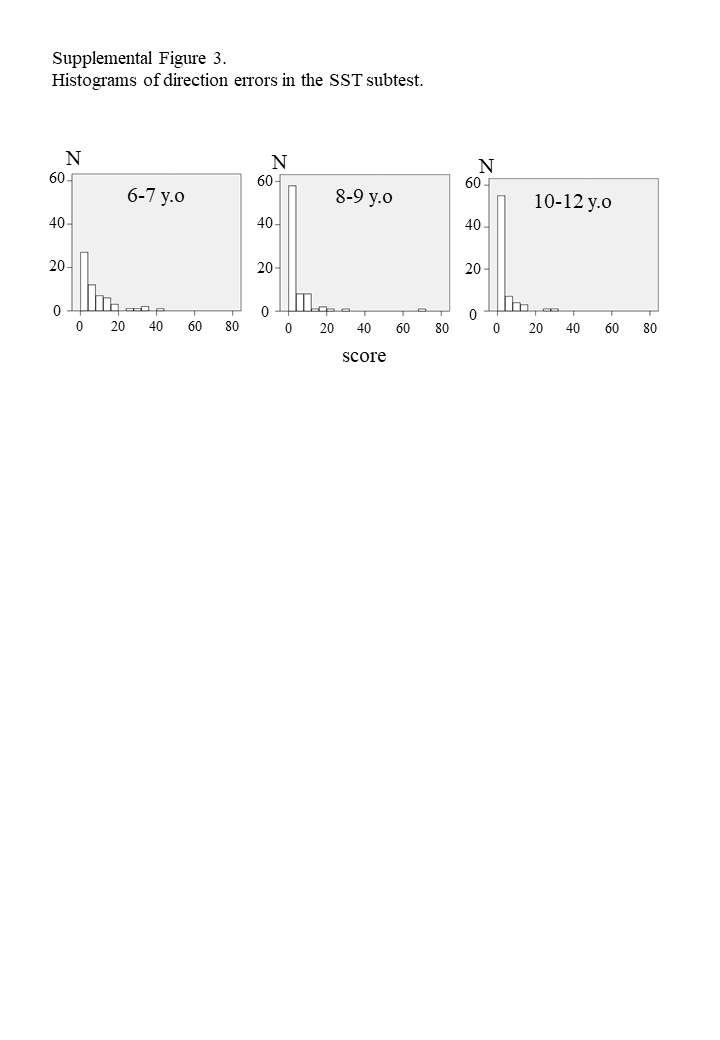

Supplement: Supplementary file 3 [file Image_3.TIF]
